# Optimizing a *C. elegans* whole organism screen biased for chemicals that target the nematode clade specific receptor EAT-2

**DOI:** 10.17912/micropub.biology.001814

**Published:** 2025-10-08

**Authors:** Henry Atemnkeng Nvenankeng, Jim Goodchild, Philippa Harlow, Vincent O'Connor, Lindy Holden-Dye

**Affiliations:** 1 University of Southampton, Southampton, England, United Kingdom; 2 Syngenta (United Kingdom), Jealott's Hill, England, United Kingdom

## Abstract

Pesticides are important resources in the control of pests and pathogens of plants and animals. Unfortunately, there are concerns around their broad impacts on non-target organisms and the environment. In this study we optimize a platform biased for the nicotinic acetylcholine receptor EAT-2 , a physiological regulator of feeding in *C. elegans .* We show that by screening for chemicals that inhibit *C. elegans* pharyngeal pumping in *lev-1 ( x427 )* , we can identify lead compounds that modulate EAT-2 function. This provides a motivation for further studies to investigate the role of EAT-2 in parasitic nematodes and their potential as a target for novel nematicides.

**Figure 1. A comparison of the dose-dependent effects of cholinergic compounds on C. elegans behaviors in WT, eat-2(ad465) and lev-1(x427) resolves EAT-2 selective effects.**
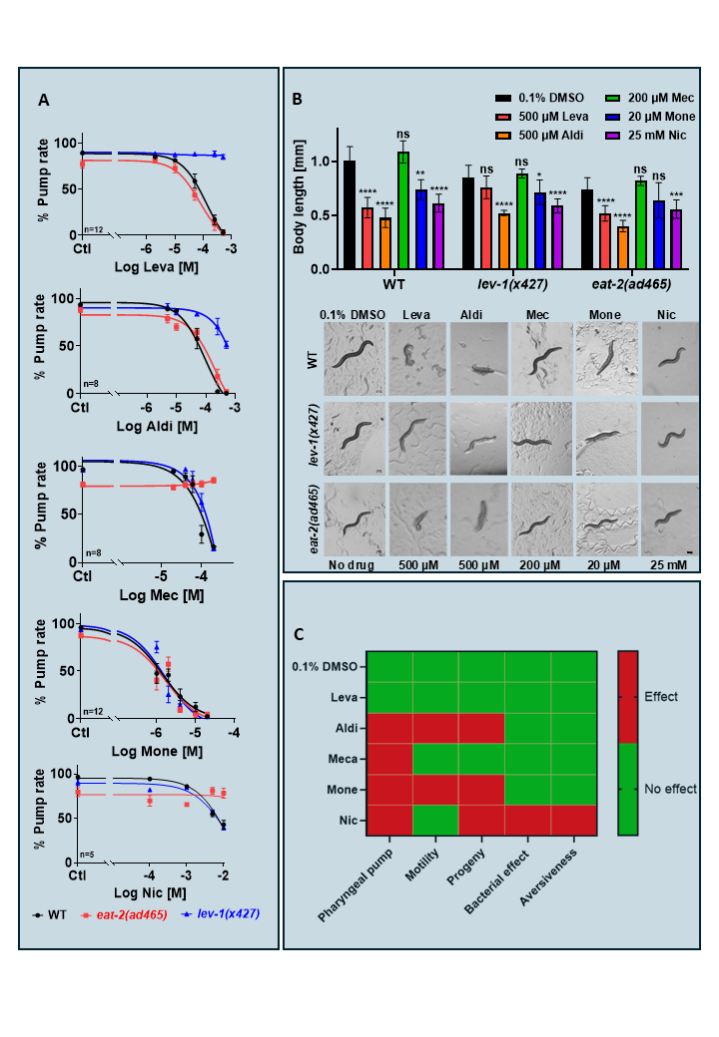
**A.** The concentration-dependent effects of cholinergic compounds levamisole (Leva), aldicarb (Aldi), mecamylamine (Mec), monepantel (Mone) and nicotine (Nic) on pharyngeal pumping (pump rate) of WT (●black), *eat-2* (■red) and *lev-1* (▲blue) worms. A 24 hour chronic exposure of nematodes to these chemicals induced concentration and strain dependent effects on pharyngeal pumping. 0.1% DMSO (control) had no effect on behaviors. At maximal concentrations, levamisole and aldicarb completely abolished feeding in WT and *eat-2* mutants, with a complete and partial resistance in *lev-1* mutants, respectively. Mecamylamine fully inhibited pharyngeal pumping in WT and *lev-1* mutants, but *eat-2* mutants were fully resistant. Monepantel elicited complete inhibition in all strains. In contrast, the inhibitory effect of nicotine was *eat-2* dependent as these mutants were resistant to nicotine induced pump inhibition observed with WT and *lev-1* mutants. **B.** The effects of the compounds on body wall muscle were assessed by measuring body length from images captured of worms exposed to the drugs for 24 hours (Image J). Levamisole and aldicarb completely paralyzed WT and *eat-2* mutants but *lev-1* mutants showed full or partial resistance to the drug induced effect, respectively. Mecamylamine did not paralyze exposed worms, monepantel spastically paralyzed them and nicotine induced a modest reduction in motility. Scale bar, 100 μm. **C.** A phenotypic readout of drug effects on *lev-1* mutants. A binary scale was used to quantify the drug induced responses 24 hours post exposure. Ligands were tested at maximal concentrations: aldicarb 500 µM, levamisole 500 µM, mecamylamine 200 µM, monepantel 20 µM and nicotine 25 mM. Statistical analyses were performed with GraphPad Prism 9 and the data are shown as mean + SEM. The analysis corresponds to the absence or presence of drug and significance tested by two way ANOVA with Bonferroni's multiple comparison ( ^ns^ p>0.05,*p<0.05 **p<0.01,****p<0.0001)

## Description

Nematicides are an important group of pesticides used to control parasitic nematodes of plants and animals, however, there are several concerns around their environmental impact, safety and sustainability (Desaeger et al., 2020). Demonstrating that a pesticide does not cause adverse effects to humans, the environment and is within reasonable economic and social costs, justifies that the said pesticide meets desired qualities for production and marketing (US EPA). There have been several approaches used to find novel chemicals that are effective and void of the aforementioned concerns. In the context of nematode control, studies in parasitic nematode pharmacology have used C *aenorhabditis elegans* as a useful tool in high-throughput anthelmintic drug discovery assays (Burns et. al 2015). By using *C. elegans* as a model system, researchers have improved on the pace of drug screening assays while focusing on drug selectivity by implementing forward and reverse genetics to identify new anthelmintic targets (Mathew et al., 2016). *C. elegans* is a powerful genetic tool that has played a significant role in defining the mode of action for anthelmintics and nematicides (Holden-Dye and Walker, 2014; Burns et al., 2015b, 2017). More targeted approaches to nematicide discovery are exploring the nematode nervous system for target sites like ion channels and receptors that have been underexploited for their pharmacological potentials (Holden-Dye and Walker, 2014).

A key factor in phenotypic assays is to establish a specific endpoint phenotype. Several behavioral experiments after exposing *C. elegans* to toxicants rely on phenotypes like motility, pharyngeal pumping, head thrash, body bends, forward/reversal movements, omega/U-turn, chemotaxis, and aversiveness (Sobkowiak et al., 2011). This method's paper describes the rationale for setting up the screening platform and using pharyngeal pumping as our primary endpoint phenotype. In *C. elegans ,* the cholinergic receptor EAT-2 , drives pharyngeal pumping and has been reported to be a potential drug target for mitigating parasitic nematodes (McKay et al., 2004; Choudhary et al., 2020). This study demonstrates how we leveraged pharyngeal pumping in a model hopping approach to investigate a novel target for parasitic nematode control. Specifically, we optimized the use of *C. elegans* in a whole organism assay to screen compound libraries and find selective modulators of EAT-2 . We hypothesize that pharmacologically modulating EAT-2 will disrupt pharyngeal muscle function, impede feeding and starve parasitic worms.

Prior studies that used *C. elegans* as model organisms in screens for novel anthelmintics or nematicides demonstrated the importance of using appropriate genetic backgrounds (Burns et al., 2015a). To achieve our aim, we used *lev-1 ( x427 )* null mutants of the levamisole-sensitive (L-type) receptor in the body-wall muscle (BWM) as our genetic background. This mutant is viable and has no phenotypic disadvantages compared to WT worms. It was selected for the study because we needed to eliminate confounding cholinergic inputs from the BWM that could introduce false positive results in our screen for modulators of pharyngeal pumping. Izquierdo et al. (2022) observed that over activating the BWM L-type receptor paralyzed wild-type worms and disrupted pharyngeal muscles function, suggesting a cross-communication between BWM and pharyngeal muscle, i.e., hypercontraction of BWM in response to cholinergic agonists indirectly causes an inhibition of pharyngeal pumping. This interpretation is supported by the observation that levamisole fails to inhibit pharyngeal pumping in *lev-1 ( x427 )* loss of function mutants (Izquierdo et al., 2022).

We characterized the chronic effects of 24 hour exposure to the selected drugs; aldicarb, levamisole, nicotine, mecamylamine and monepantel on *lev-1 ( x427 )* mutants and then compared the data with those from WT and *eat-2 ( ad465 )* mutants. These selected ligands were assessed for their effects on; pharyngeal pumping. In parallel, we assessed their effects on body wall muscle contraction, motility, progeny development and aversiveness. To ensure the effects observed were not due to an effect of the compounds on the bacterial lawn rather that a direct effect on *C. elegans* behavior we also tested their effects on *Escherichia coli* , growth (Table 1).

In agreement with published observations, levamisole, agonist of the L-type receptor in *C. elegans* BWM had no effect on *lev-1 ( x427 )* mutants compared to WT and *eat-2 ( ad465 )* mutants where pharyngeal pumping was totally inhibited and the worms were paralyzed ( Figure 1A & B). Pharyngeal pump inhibition observed with WT and *eat-2 ( ad465 )* worms is due to hypercontraction of the BWM ( Figure 1B ) (Izquierdo et al., 2022) and in agreement with the observation that levamisole is not a modulator of EAT-2 (Choudhary et al., 2020). A similar result was observed with aldicarb, a cholinesterase inhibitor that disrupts cholinergic pathways in the BWM and pharynx, resulting in paralysis and pharyngeal pump inhibition. Aldicarb inhibits ACh breakdown at the neuro-muscular junctions of BWMs and the pharynx, chronically elevating ACh levels. This induces a spastic paralysis, immobilizes the worms and inhibits pharyngeal pumping (Mahoney et al., 2006; Izquierdo et al., 2023). Monepantel induced paralysis in WT, *eat-2 ( ad465 )* and *lev-1 ( x427 )* mutants also resulting in an indirect inhibition of pharyngeal pumping due to BWM contraction ( Figure 1A & B). Previously, monepantel has been reported as an agonist of ACR-23 receptor of the BWM, inducing a spastic paralysis (Rufener et al., 2013). Interestingly, mecamylamine and nicotine had inhibitory effects on pharyngeal pumping void of paralysis and although nicotine induced a modest inhibitory effect on motility, this did not impact pharyngeal pumping in *eat-2 ( ad465 )* mutants, suggesting EAT-2 to be a target for the drug. Mecamylamine had a more selective effect as the only phenotypic disruption observed was pharyngeal pump inhibition in WT and *lev-1 ( x427 )* mutants. Mutants of *eat-2 ( ad465 )* were resistant to this drug-induced inhibition, suggesting it to be a target of the drug. In recombinant assays, nicotine and mecamylamine are reported to be an agonist and antagonist of EAT-2 , respectively (Choudhary et al., 2020).

We optimized the screening platform to investigate modulators of the nAChR EAT-2 , and showed that it was also suitable for obtaining secondary data of drug effect on egg laying and development, motility, aversiveness and bacterial growth. We demonstrated that pumping in *C. elegans* could be directly inhibited by modulating EAT-2 function, as seen with nicotine and mecamylamine or indirectly as in the case of levamisole, monepantel and aldicarb. Care must be taken when interpreting the data as pump inhibition does not directly imply an EAT-2 effect. Lead compounds identified must be tested in other paradigms to ensure that a true interaction exists with the target. EAT-2 receptor function can be compromised through overactivation by agonists, resulting in an activation block or by an antagonist, in which case it blocks the receptor activation, keeping pharyngeal muscles extendedly relaxed.

## Methods

***C. elegans* maintenance**

*C. elegans ,* N2 (WT, Bristol strain), *eat-2 ( ad465 )II* and *lev-1 ( x427 )IV* mutants were obtained from the Caenorhabditis Genetics Centre (CGC), grown and maintained under standard conditions (Brenner, 1974). Synchronized *C. elegans* L4 worms were identified by the crescent-shaped, vulva saddle clearing made by the maturing vulva (Divekar et al., 2021), and picked onto OP50 seeded plates, incubated overnight prior to performing behavioral assays with them as young adults (L4+1).

**Drug stocks**

Aldicarb, levamisole, nicotine, mecamylamine were purchased from Sigma-Aldrich Company Ltd, UK. Monepantel was provided by Syngenta, UK. Nicotine, levamisole, aldicarb and mecamylamine were dissolved in sterile double-distilled water to make stock solutions. Monepantel was dissolved in 100% DMSO and tested at 0.1% (v/v) to negate the adverse effects of DMSO on multiple aspects of *C. elegans* biology (Calahorro et al., 2021). All drug stocks stored at 4 ^ᵒ^ C were used within a month of preparation.

**Drug screening plates**

The protocol developed to interrogate EAT-2 function was setup in 24-well plates. 3 ml of molten NGM was added into each well and left in the laminar flow for 30 minutes to solidify. Control wells were seeded with 25 µl of OP50 only or laced with 3 µl of the vehicle, DMSO. To test nicotine, levamisole, aldicarb, mecamylamine and monepantel, 3 µl from stock concentrations was added to 25 µl of OP50 and then used to seed wells. The drop of OP50 was spread over the whole surface of NGM in each well and left for 30 minutes in the laminar flow to dry. These plates were then stored overnight at 4 ^ᵒ^ C with the expectation that the drug equilibrates over the entire 3 ml NGM. Before experimenting, plates taken out of the fridge were set on the bench for at least 30 minutes to equilibrate to the room temperature (rtp). Three L4+1 worms were picked into each well and incubated for 24 hours at rtp. The following day, phenotypic observations on pharyngeal pumping, motility, progeny development, averseness and potential bactericidal effects were scored (Table 1). These observations were made using the Nikon SMZ800 binocular dissecting microscope (60x)

**Table 1. Qualitative and quantitative measurements of drug-induced effects on *C. elegans***

**Table:** 

**Phenotype**	**Observation and Quantification**		**Score ( 0 or 1)**
Pharyngeal pumping	One pharyngeal pump is a complete cycle of one forward and backward movement of the grinder. Using a counter and an electronic stopwatch, pump rates were measured for one minute per worm.		Pump <200 = 1
Motility	Motility effect was quantified mainly by looking for paralysis. Worms were considered paralyzed if they failed to move when prodded 3 times with an aluminium picking needle. Measuring the body length of paralyzed worms informed on the type of paralysis (spastic or flaccid).		No movement = 1
Progeny	The number of eggs and juveniles were counted 24 hours post-incubation.		Eggs and Juveniles < 150/3 = 1
Aversiveness	Worms crawling off plates were scored for drug aversiveness by counting the number of worms on the food lawn 24 hours post-incubation.		< 2/3 worms = 1
Bacterial effect	Based on visual observations, drugs that affected the establishment of the food lawn compared to the control were considered to have an effect on bacterial growth.		Unestablished bacteria lawn = 1

Scoring was binary. There was either an effect (1) or no effect (0).

**Dose-time response assays**

Dose-time response assays for hit compounds that emerged from the screen were performed on 6-well plates containing NGM. OP50 was laced with a range of different drug concentrations and then used to seed NGM plates. Drug dose induced effects on pharyngeal pumping was recorded at scheduled times over 24 hours.

**Statistical analysis**

Data was analyzed using GraphPad Prism 10 and displayed as mean ± standard error of the mean (SEM) of the number of worms used for each assay. The number of worms accessed for each experiment was specified on the corresponding figure. Statistical significance was assessed using two way ANOVA followed by a post hoc analysis with Bonferroni corrections.
